# MAKING BUILT ENVIRONMENT MORE AGE-FRIENDLY -- FIRST TASK OF UNIVERSITY OF COLORADO AGE-FRIENDLY TASKFORCE

**DOI:** 10.1093/geroni/igae098.4237

**Published:** 2024-12-31

**Authors:** Kathryn Nearing, Jodi Waterhouse

**Affiliations:** University of Colorado Anschutz Medical Campus, Denver, Colorado, United States; CU Anschutz Multidisciplinary Center on Aging, Aurora, Colorado, United States

## Abstract

When the Age-Friendly University (AFU) Taskforce at the University of Colorado Anschutz Medical Campus initiated strategic planning in 2023, the built environment – an antecedent condition for achieving AFU priorities – emerged as priority #1. The taskforce tailored an existing tool available from University of Massachusetts-Boston to align the built-environment assessment with the broader mission-focused areas of CU Anschutz – one of the first four academic medical campuses in the world to receive the AFU designation. During April 2023, each taskforce representative paired with an older adult community member; each dyad was assigned a campus zone to assess using the adapted Age-Friendly Inventory and Campus Climate Survey. The community member provided first-hand accounts of how they experienced aspects of the built environment, while the taskforce representative recorded observations. Once compiled, the taskforce reviewed data and identified the following areas of concern: wayfinding supports (e.g., signs and campus maps -- printed, online); parking kiosk accessibility; distances (e.g., from parking to campus buildings); campus building and restroom accessibility. Taskforce members presented results and recommendations to the Chancellor, campus architect, Vice Chancellor for Facilities, and Communications team. Through campuswide collaborations that expanded to include parking managers and the campus shuttle service, issues with campus maps, parking kiosks, crosswalks, walking distances, and building/restroom accessibility are being addressed, with a progress report provided to the Chancellor in July 2024. This work is essential. The built environment fundamentally influences campus accessibility across the lifespan and the age-inclusivity of events, classes, research studies and healthcare services.

